# Selection of important variables by statistical learning in genome-wide association analysis

**DOI:** 10.1186/1753-6561-3-s7-s70

**Published:** 2009-12-15

**Authors:** Wei (Will) Yang, C Charles Gu

**Affiliations:** 1 Division of Biostatistics, Washington University School of Medicine, 660 South Euclid Avenue, St. Louis, Missouri 63110, USA; 2 Department of Genetics, Washington University School of Medicine, 660 South Euclid, St. Louis, Missouri, Missouri 63110, USA

## Abstract

Genetic analysis of complex diseases demands novel analytical methods to interpret data collected on thousands of variables by genome-wide association studies. The complexity of such analysis is multiplied when one has to consider interaction effects, be they among the genetic variations (G × G) or with environment risk factors (G × E). Several statistical learning methods seem quite promising in this context. Herein we consider applications of two such methods, random forest and Bayesian networks, to the simulated dataset for Genetic Analysis Workshop 16 Problem 3. Our evaluation study showed that an iterative search based on the random forest approach has the potential in selecting important variables, while Bayesian networks can capture some of the underlying causal relationships.

## Background

Complex diseases such as coronary heart disease (CHD) are results of failures in complex biological systems. Multiple factors (genetic and environmental) are involved in the etiology; and the disease outcomes most likely reflect the interactions of the factors involved at different biological levels and in varying context. This has led to the new "global" approach to genetic studies of common diseases, including the most recent genome-wide association studies (GWAS); it has also brought tremendous analytical challenges [1]. For example, methods depending solely on *p*-values from testing individual single-nucleotide polymorphisms (SNPs) may not be enough to identify culprit variants in a vast sea of SNPs [2]. On the other hand, although considered crucial in the genetic composition of diseases, interactions are seldom analyzed directly in GWAS studies. Even pair-wise interactions among SNPs are already computationally strenuous [3].

Our investigation showed that the approach of statistical learning (i.e., selecting sets of variables by repeated learning from examples [4]) seemed promising in dealing with high-dimensional problems, especially in the presence of interactions. In particular, several well known statistical learning methods demonstrated their capabilities in handling the complexity of GWAS in different scenarios and at different levels. For example, random forest (RF) [5] performed quite well in small datasets and simulation studies [6,7]. It seems that without explicitly modeling the interactions, RF can rank predictors reasonably well by counting in their joint effects. On the other hand, Bayesian networks (BNT) analysis seems capable of capturing biologically meaningful interactions among a group of factors involved in a complex manner in common diseases [8,9]. However, neither method seem suitable for being directly applied to GWAS data, which now typically have over 500 k variables. The simulated data set from Genetic Analysis Workshop (GAW) 16 Problem 3 provides an opportunity for identifying breaking points of these learning methods and for evaluating their extensions for handling extremely high-dimensional GWAS data.

## Methods

### GAW16 Problem 3 data set

The simulated data of GAW16 Problem 3 is based on the Framingham Heart Study pedigrees of 6,476 individuals, and real genome-wide SNPs typed using two Affymetrix platforms (500 k and 50 k arrays) [10]. This evaluation study is performed on a random sample of 1,117 independent individuals selected from the familial dataset. There are two simulated cardiovascular phenotypes, the binary myocardial infarction (MI) and the continuous endophenotype coronary artery calcification (CAC). MI is directly affected by two groups of interacting factors (one among SNPφ_1_, smoking, and CAC; the other SNP φ_2 _and CAC). CAC is directly affected by five SNPs (τ_1 _and τ_2 _interact, τ_1 _has weak marginal effect, τ_2 _a measurable additive effect; τ_3 _and τ_4 _interact, but none with detectable marginal effect; τ_5 _displays heterosis, i.e., only the heterozygous genotype is protective). Other simulated risk factors include high-density lipoprotein (HDL), total cholesterol (CHOL) and triglyceride (TG) levels, and the age of the subjects. Longitudinal data of phenotypes were simulated for three time points in 200 replicate datasets. We used only the second observations of phenotypes (with '2' appended after variable names, e.g., CAC2) and the 50 k genotypes.

### RF analysis

RF originates from the classification and regression trees (CART) [5,7]. It consists of a collection of trees, each grown on a bootstrap sample of observations, and at each node of a tree, small random subset of predictors is searched for the best split. Prediction of RF is made by aggregating predictions from all trees in the forest. Unbiased generalization error is generated without requirement of an extra testing sample. It provides measures for each variable's predictive importance. Because the importance measure for a variable entails interaction effects with other factors, there may be no need to explicitly model each possible interaction terms repeatedly. This feature makes it most suitable for large-scale studies such as GWAS.

However, direct application of RF to 500 k or even 50 k SNPs may not be feasible. Because of the extremely high dimensionality, including all SNPs in a single RF analysis can lead to "fitting to noises". We devised a new procedure to limit the number of variables that are piped to RF in an iterative manner. Each variable in the large data set could be evaluated many times with different groups of other variables. Globally important variables could be selected after many iterations. At the end, our RF procedure returns a very small set (~15 for the current study) of predictors from all input variables (GWAS SNPs and other covariates), which have high importance and jointly give pretty good prediction rate by RF.

This procedure is tested on CAC2 levels and the 50 k SNPs data. We consider only the first ten replicate data sets due to time constraint (each with ≤ 1,000 iterations), and we are interested in how many times the true risks were captured in the returned set of predictors. We used the R package randomForest for deriving RFs.

### BNT analysis

BNT is a graphical model used to learn structure (joint multivariate probability distribution) of a set of random variables that reflects relationships of dependence and conditional independence among them [8,9]. In a BNT, variables are represented as vertices (nodes) and dependencies as arcs (or edges) between the variable nodes. The directions of edges indicate the directions of dependencies favored by the observed data, although they do not necessarily imply causality. We applied BNT to the set of known predictors for the binary MI event and its endophenotype CAC to see whether the method can reliably "learn" important relationships among all relevant variables. Four scenarios were considered. Scenario 1: test includes MIevent2, CAC2, and true predictors: smoke, age, HDL, CHOL and the seven risk SNPs; Scenario 2: includes all variables in Scenario 1 and seven random SNPs that are not in linkage disequilibrium (LD) with any risk SNPs; Scenario 3: includes all variables in Scenario 1 and seven random SNPs that are in LD with the risk SNPs; Scenario 4: includes all variables in Scenario 1 and 50 random SNPs. We applied 100-fold bootstrapping to assess the stability of derived relationships. Results were averaged over the 200 replicate data sets.

## Results

### RF analysis

**Figure 1:**
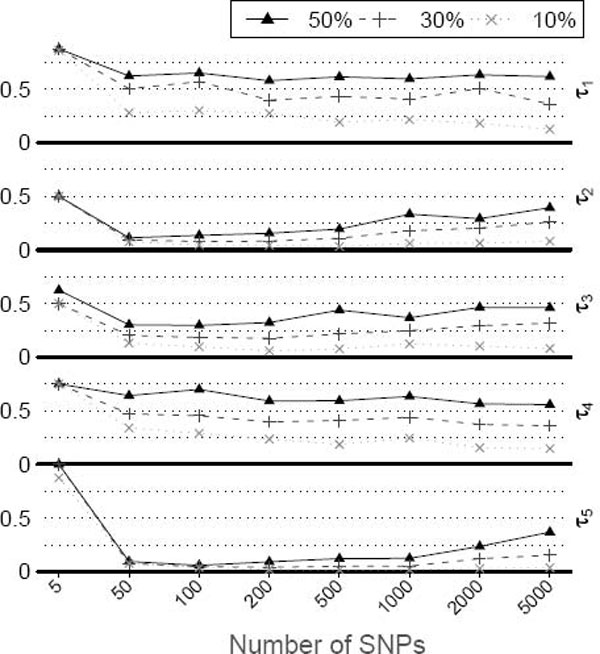
**Rank of risk SNPs in random forest as noise level increases**. The five risk SNPs (τ_1_-τ_5_) for CAC were tested with 3 environment factors and different numbers of noise SNPs (see text). At each level of noises, the test was repeated 100 times. We define relative rank of a variable to be the rank of variable importance normalized by the total number of predictors. Lower value indicates the variable is easier to be detected by random forest. The plot shows the quantiles of relative rank for the five risk SNPs. The 3 kinds of curves represent 50^th^, 30^th ^and 10^th ^quantiles, respectively (marked as "50%", "30%" and "10%" in the plot).

Before testing our new procedure, we first performed an experiment to examine how too many noise SNPs could result in the RF "fitting to noise". Original RF was applied in tests that included five risk SNPs of CAC, three environment covariates, and different numbers of randomly selected (noise) SNPs. This was repeated in the first 100 replicate datasets and results are summarized in Figure 1, where the "relative rank" of each risk SNP is plotted against the number of noise SNPs. The measure is simply the importance rank for that SNP divided by the total number of predictors, and should tell if the SNP could be picked out at a certain cut-off. It is clear that τ_1 _and τ_4 _are always difficult to detect (median relative rank > 0.5). When the noise SNPs are not many, they could easily be found among the top important predictors. As the noise level increases, it becomes difficult to detect them. This shows the necessity to limit the number of variables in the RF analysis, as we proposed to do in the iterative procedure.

We then applied the new iterative procedure that limits number of SNPs fed to the RF. The method almost always identified the three major risk factors, CHOL, HDL, and age (nine, ten, and ten times out of ten replicates, respectively), and detected consistently one CAC risk SNP τ_5 _(five times out of ten replications) that has substantive marginal effect in the true model. It seems that the performance of the procedure was less than optimal in detecting "pure" interactions because none of the other four risk SNPs was identified.

Finally, we compared the modified RF analysis with the original RF that uses all GWAS SNPs (41,006) and eight covariates as predictors, to see whether the iterative procedure indeed performed any better. The three covariates were still always ranked with top importance in the RF test. However, the ranks of the five risk SNPs were generally very low. Only two SNPs were ever found among the top 100 predictors. Once τ_2 _was ranked at 85 and another time τ_5_was ranked at 69. In contrast, using our new procedure τ_5 _was detected five times, with a ranking of 19 or better.

### Bayesian network tests

Many real relationships, especially those between CAC, smoke, and MI event were recovered by BNT analysis, while many others were not. In Table 1, we show results of bootstrapping analyses that assess the stability of learned relationships, where φ_1_, φ_2 _and τ_1_-τ_5 _are true risk SNPs, and n1, n2, ..., denotes noisy SNPs included in the learning dataset. The analysis was repeated in all 200 replicate datasets and averaged results are displayed in Table 1. In all four scenarios, relationships between smoke and MI, CAC and MI, CHOL and CAC are most reliably detected (>50%). Other environment risk factors and the genetic factors also show appreciable confidence (over or close to 20%), while noise SNPs generally have averaged below 10%. Note that some important relationships involving τ_5 _(with MI and CAC) also enjoyed the best reproducibility (37%) compared with those for other risk SNPs. While LD among noise and risk SNPs seemed irrelevant, the reproducibility quickly deteriorates as the number of noise SNPs increases. It becomes hard to discriminate risk SNPs from noise when we included over 50 random SNPs.

**Table 1:** Bootstrapping results of detected edges from predictors to phenotypes

	Scenarios							
	
	Original		+ 7 LD free SNPs		+ 7 SNPs with LD		+ 50 random SNPs	
				
Predictors and SNPs	MIevent2	cac2	MIevent2	cac2	MIevent2	cac2	MIevent2	cac2
cac2	**0.575^a^**	0.000	**0.603**	0.000	**0.626**	0.000	**0.541**	0.000
smoke2	**0.600**	*0.264*	**0.557**	*0.274*	**0.603**	*0.252*	**0.466**	*0.238*
age2	*0.218*^b^	*0.253*	0.196	**0.343**	0.181	**0.365**	*0.296*	*0.250*
chol2	0.173	**0.670**	0.176	**0.692**	0.174	**0.691**	0.122	**0.713**
hdl2	0.162	**0.362**	*0.265*	*0.286*	*0.284*	*0.265*	0.124	*0.275*
sex	*0.257*	*0.247*	*0.200*	*0.206*	0.183	*0.205*	0.092	0.099
τ_1_	*0.213*	*0.215*	0.146	0.126	0.144	0.122	0.063	0.067
τ_2_	*0.230*	*0.236*	0.171	0.154	0.175	0.156	0.102	0.059
τ_3_	0.192	*0.283*	0.136	0.134	0.113	0.135	0.090	0.085
τ_4_	*0.209*	*0.270*	0.179	0.185	0.179	*0.204*	0.057	0.084
τ_5_	*0.218*	*0.252*	0.195	**0.371**	0.168	**0.321**	0.107	0.084
τ_6_	*0.245*	0.169	*0.204*	0.106	*0.228*	0.096	0.099	0.076
τ_7_	*0.236*	0.158	0.182	0.136	*0.207*	0.133	0.110	0.063

n1			0.067	0.063	0.079	0.106	0.040	0.036
n2			0.083	0.093	0.005	0.005	0.012	0.010
n3			0.041	0.051	0.127	0.097	0.013	0.016
n4			0.079	0.064	0.015	0.007	0.033	0.042
n5			0.064	0.065	0.068	0.061	0.007	0.015
n6			0.040	0.034	0.113	0.143	0.022	0.032
n7			0.032	0.032	0.022	0.019	0.028	0.018

^a^Bold font indicates relationship found in >30% of the 200 replicates by bootstrapping.

^b^Italic, underlined font indicates relationship found in >20% (≤ 30%) of the 200 replicates by bootstrapping.

## Discussion

Extensions of RF have been proposed and applied to gene expression data, aimed at identifying variables important to the trait of interest [11]. These approaches start from the whole set of predictors and gradually decrease the number of predictors fed to RF by discarding the worst performers at each iteration. For application of RF to GWAS, the study by Schwarz et al. presented at GAW15 [12] is particularly interesting. They repeatedly grow 155 RFs with 5,000 variables each and averaged the importance score to get global importance, then performed forward elimination to select best predicative model. In the current study, we showed that including too many noise SNPs in single RF runs can lead to compromised power. We propose a new procedure that limits the number of predictors piped into RF at each iteration to control fitting to noise. Comparison with original RF analysis including all GWAS SNPs seemed to support this strategy. However, the new procedure did not perform as well as we expected. We suspect that the suboptimal performance was partly due to small sample size or the fact that effects of SNPs are low, and partly due to the peculiar distribution of CAC phenotype (which has lots of values "0", and other values scattered along the positive axis, and thus departs quite far from normal distribution, which may make using variance and mean square error an ineffective way to measure the performance of regression trees).

Our evaluation of BNT showed that it is ill-performed when the noise level is too high. It may be best applied after initial filtering of candidate SNPs and used it to facilitate the interpretation of results and forming new hypothesis.

## Conclusion

We evaluated two statistical learning methods to GWAS analysis using simulated data from GAW16 Problem 3. We showed that including too many noise SNPs in the analyses may seriously affect their performance. By limiting number of SNPs fed to RF in a new iterative procedure, our method out-performed direct application of RF to the whole GWAS dataset. BNT analysis recovered some but not all relationships and its performance also deteriorated as more noise SNPs were included in the analysis.

These findings demonstrate that the applications of advanced statistical learning methods to GWAS require careful consideration on how to limit inclusion of potential noise SNPs. Further studies are needed to investigate issues related to sample sizes and false discovery rate of these methods.

## List of abbreviations used

BNT: Bayesian networks; CAC: Coronary artery calcification; CART: Classification and regression trees; CHD: Coronary heart disease; CHOL: Cholesterol; GAW: Genetic Analysis Workshop; GWAS: Genome-wide association studies; HDL: High-density lipoprotein; LD: Linkage disequilibrium; MI: Myocardial infarction; RF: Random forest; SNP: Single-nucleotide polymorphism; TG: Triglyceride.

## Competing interests

The authors declare that they have no competing interests.

## Authors' contributions

WY developed method, performed analysis, and drafted the manuscript. CCG developed the concept, participated in analysis, interpreted results, revised the manuscript critically, and gave final approval for publication. All authors read and approved the final manuscript.
